# Data on pain coping strategies and their association with quality of life in people with Parkinson’s disease: A cross-sectional study

**DOI:** 10.1016/j.dib.2022.108288

**Published:** 2022-05-17

**Authors:** Tino Prell, Jenny Doris Liebermann, Sarah Mendorf, Hannah M. Zipprich

**Affiliations:** a Department of Geriatrics, Halle University Hospital, Halle, Germany; b Center for Healthy Aging, Jena University Hospital, Am Klinikum 1, Jena 07747, Germany; c Department of Neurology, Jena University Hospital, Am Klinikum 1, Jena 07747, Germany

**Keywords:** Pain, Coping, Self-efficiency, Parkinson disease, Quality of life

## Abstract

This article presents data about coping with pain and health-related quality of life from 52 patients with Parkinson’s disease (PD) (without PD dementia). Coping was assessed using Coping Strategy Questionnaire (CSQ), including active/passive and cognitive/behavioral coping strategies and the felt efficacy of the coping strategies used. In addition, common PD specific assessments were recorded. For pain rating the corresponding items from the Short-Form-36 were used. The dataset allows determining factors related pain and coping in PD. The dataset can be utilized by clinicians, academics and pharmacists for further research and reference purposes. The data presented herein is associated with the research article “Pain coping strategies and their association with quality of life in people with Parkinson's Disease: a Cross-Sectional study” [1] and available on Dryad, Dataset 10.5061/dryad.2280gb5s7.

## Specifications Table

**Table:** 

Subject	Geriatrics and Gerontology
Specific subject area	Health Services Research
Type of data	Table
How the data were acquired	Survey using the Coping Strategy Questionaire (CSQ). Data from 52 patients with PD and informed consent were collected (consecutive sampling) spending their time on the neurological ward in the Clinic of Neurology at the Jena University Hospital, Jena, Germany. Statistical analyses were performed using SPSS version 25.0 (IBM, New York, NY, USA) and R version 3.6.2 (R Foundation for Statistical Computing, Vienna, Austria), with a p-value of <0.05 indicating statistical significance.
Data format	Raw Analysed
Description of data collection	All subjects were treated in the PD multimodal complex therapy. Assessments were collected at the start of the inpatient stay. Patients were included for the following reasons: deep brain stimulation evaluation, increasing fluctuations, increasing off-phases, freezing and gait deterioration. Patients with significant cognitive impairments based on the Montreal Cognitive Assessment (MoCA ≤21) were excluded.
Data source location	Department of Neurology, Jena University Hospital Am Klinikum 1, 07747 Jena, Germany
Data accessibility	Repository name: T. Prell, Pain Coping Strategies and their Association with Quality of Life in People with Parkinson’s disease: a Cross-Sectional Study, Dryad, Dataset, (2021).
	Data identification number: 10.5061/dryad.2280gb5s7 Direct link to the dataset: https://datadryad.org/stash/dataset/doi:10.5061/dryad.2280gb5s7
Related research article	T. Prell, J.D. Liebermann, S. Mendorf, T. Lehmann, H.M. Zipprich, Pain coping strategies and their association with quality of life in people with Parkinson's disease: A cross-sectional study, PLoS One 16(11) (2021) e0257966. [1]

## Value of the Data

• The data reported in this article provide information about coping strategies of patients with PD.
• The data reported in this article can be used to investigate pain management and its influencing factors in people with PD.
• The data reported in this article can be used for general analyses about people with PD, as general and disease-specific data are given.
• The data reported in this article can be used by clinicians and academia for further research as well as reference.

## 1 Data Description

The data article reports demographical and clinical data as well as data about coping and health-related quality of life (Table 1). Data from 52 individuals have been provided (age 74.4, SD 6.6, 38.5% female). About 65% (n = 34) were in Hoehn & Yahr stage 3, which was followed by stage 2 (n = 8) and stage 4 (n = 8), and two patients showed stage 1. Pain was analyzed using the related SF-36 items 21 and 22. Two/fifth were in no (n = 5), very mild (n = 5), or mild (n = 10) physical pain, and four/fifth suffered from moderate (n = 14), severe (n = 14), or very severe (n = 3) pain in the past month. The impact of pain on normal work was described by 51% as not at all (n = 6), a little (n = 10), and moderately (n = 10). Of note, conversely, 49% described a strong (n = 19) and extreme (n = 6) impact on work (SF-36 item 22). The items and scales of the CSQ are presented in the data set. Overall, 33 (64%) utilized active coping strategies and 19 (36.5%) utilized passive coping strategies based on the combination of the scales on CSQ factors (n = 14, 26.9%). Descriptive statistics of the sample are presented in Tables 2 and 3.

**Table 1:** Variables description.

Variable	Type/ unit	Description	Explanation
Age	Numerical years	Individual age, grouped	
Sex	Nominal	gender	1 male; 2 female
Housing_situation	Categorical	Housing situation	1 single; 2 not alone
Disease_duration	Numerical years	Disease duration, grouped	
HY	Ordinal	Hoehn & Yahr stage	
MDS-UPDRS_subscore_lll	Numerical	Movement Disorder Society-sponsored revision of the Unified Parkinson's Disease Rating Scale III, grouped	
NMSQ	Numerical	revised nonmotor symptoms questionnaire, grouped	
MOCA	Numerical	Montreal Cognitive Assessment, grouped	
BDI_ll	Numerical	Beck Depression Inventory II, grouped	
		Coping (Coping Strategy Questionnaire (CSQ)):	
CSQ_DA_Transformed	Numerical	Diverting Attention	
CSQ_RPS_Transformed	Numerical	Reinterpreting Pain Sensations	
CSQ_CSS_Transformed	Numerical	Coping Self-Statements	
CSQ_IPS_Transformed	Numerical	Ignoring Pain Sensations	
CSQ_PH_Transformed	Numerical	Praying or Hoping	
CSQ_CA_Transformed	Numerical	Catastrophizing	
CSQ_IAL_Transformed	Numerical	Increasing Activity Level	
CSQ_IPB_Transformed	Numerical	Increasing Activity Level	
CSQ_CP_Transformed	Numerical	Control over Pain	
CSQ_DP_Transformed	Numerical	Ability to Decrease Pain	
		Short Form 36 (SF-36):	
SF36_rphysf_c	Numerical	Physical functioning	
SF36_rsocf_c	Numerical	Social functioning	
SF36_rrolef_c	Numerical	Role functioning/physical	
SF36_rrolee_c	Numerical	Role functioning/emotional	
SF36_rment_c	Numerical	Emotional well-being	
SF36_rvit_c	Numerical	Energy/fatigue	
SF36_rpain_c	Numerical	Pain	
SF36_rgenh_c	Numerical	General Health	
SF36_rhchange_c	Numerical	Health change	
SF36_item_pain	Ordinal	How much bodily pain have you had during the past 4 weeks?	1 none; 2 very mild; 3 mild; 4 moderate; 5 severe
SF36_item_painADL	Ordinal	How much did pain interfere with your normal work?	1 not at all; 2 a little bit; 3 moderately; 4 quite a bit; 5 extremely

**Table 2:** Descriptive statistics – nominal, categorical and ordinal variables.

		n = 52	
Sex (n,%)	Female	20	38.5
	Male	32	61.5
Housing situation	Alone	15	28.8
	Not alone	37	71.2
Hoehn and Yahr stage (median, IQR)		2.93	0.80
SF36 item: How much bodily pain have you had during the past 4 weeks? (median, IQR)		4	2
SF 36 item: How much did pain interfere with your normal work? (median, IQR)		3	2

**Table 3:** Descriptive statistics – numerical variables.

	n = 52	
	Mean	SD
Age (years)	74.38	6.60
Disease duration (years)	8.86	5.21
MDS-UPDRS III	32.45	14.85
NMS-Quest	11.18	4.73
Montreal Cognitive Assessment	25.15	3.09
Beck Depression Inventory II	11.53	6.28
**Coping (Coping Strategy Questionnaire (CSQ)):**	**Mean**	**SD**
Diverting Attention	40.43	19
Reinterpreting Pain Sensations	23.08	16.49
Coping Self-Statements	52.62	17.0
Ignoring Pain Sensations	41.5	22.11
Praying or Hoping	30.6	16.53
Catastrophizing	34.89	18.3
Increasing Activity Level	43.11	19.38
Increasing Activity Level	46.64	15.86
Control over Pain	42.95	27.08
Ability to Decrease Pain	41.34	26.09
**Short Form 36 (SF-36):**	**Mean**	**SD**
Physical functioning	39.41	25.01
Social functioning	56.86	27.08
Role functioning/physical	18.0	29.89
Role functioning/emotional	47.06	44.81
Emotional well-being	60.94	15.79
Energy/fatigue	44.71	15.34
Pain	45.74	25.7
General Health	39.22	14.84
Health change	30.88	23.23

## 2 Experimental Design, Materials and Methods

### 2.1 Experimental design

In this observational study, people with PD were consecutively recruited from the Department of Neurology at the Jena University Hospital between May 2019 to July 2019. This study was approved by the local ethics committee of the Jena University Hospital (4572-10/15). The participants gave their written agreement in accordance with The Code of Ethics of the World Medical Association (Declaration of Helsinki). All patients arrived at the clinic as scheduled and were enrolled in a Parkinson's-specific complex program. As part of this, multimodal therapy by specialized therapists and medication optimization took place (Multimodal Complex Treatment for PD) [2]. Assessments were collected at the start of the inpatient stay. Patients were included for the following reasons: deep brain stimulation evaluation, increasing fluctuations, increasing off-phases, freezing and gait deterioration. Patients with significant cognitive impairments based on the Montreal Cognitive Assessment (MoCA ≤21) were excluded [3].

### 2.2 Materials

We collected demographic and PD-specific data: Age, gender and living situation, Movement Disorder Society-sponsored revision of the Unified Parkinson's Disease Rating Scale III (MDS-UPDRS III) [4], the revised Nonmotor Symptoms Questionnaire (NMS-Quest) [5], and the Hoehn and Yahr staging; MoCA was utilized to assess cognitive ability. The BDI was used to assess depression (BDI-II). The Short Form 36 (SF-36) of the Medical Outcomes Study (MOS) was utilized to assess health-related quality of life [6,7]. The calculation of the SF-36 subscales was made according to the standardized algorithm based on instructions from RAND Health Care (www.rand.org/health-care/surveys_tools/mos/36-item-short-form/scoring.html). Subsequently, the items of every scale were added and newly scaled in a standard interval from 0 to 100. A value of 100 indicates the highest level of health.

The corresponding SF-36 subscale was utilized to assess pain: Item 21 (How severe was your physical pain in the past four weeks?) and Item 22 (How much did pain interfere with your normal work (including work outside the home and housework) in the past four weeks?); lower scores indicate more severe pain.

Pain coping was rated with the Coping Strategy Questionnaire (CSQ) (https://igptr.ch/wp-content/uploads/2019/09/CSQ-D.pdf). It is a commonly used, internationally validated questionnaire on pain coping strategies measuring not only active/passive but also cognitive/behavioral coping mechanisms, as well as the perceived effectiveness of the coping strategies used [8]. The CSQ-D includes 50 items of pain coping used by the patient respondent. Patients are asked to assess what they do when they are feeling pain and to select the most appropriate response. For this reason, the CSQ scales were divided into CSQ factors: active and passive pain coping strategies, and self-efficacy [9].

## Ethics Statements

This study was approved by the local ethics committee of the Jena University Hospital (4572-10/15). The participants gave their written agreement in accordance with The Code of Ethics of the World Medical Association (Declaration of Helsinki).

## Informed Consent Statement

Informed consent was obtained from all subjects involved in the study.

## Funding

TP received funding by a BMBF grant (01GY1804).

## Declaration of Competing Interest

The authors declare that they have no known competing financial interests or personal relationships that could have appeared to influence the work reported in this paper.

## Data Availability

Prell, Tino (2021), Pain Coping Strategies and their Association with Quality of Life in People with Parkinson’s disease: a Cross-Sectional Study, Dryad, Dataset, 10.5061/dryad.2280gb5 (Original data) (Dryad).

## Appendix Supplementary materials

Image, application 1

Image, application 2
