# Supplementary material for: Data on pain coping strategies and their association with quality of life in people with Parkinson’s disease: A cross-sectional study
Source: Data Brief. 2022 May 17;42:108288. doi: 10.1016/j.dib.2022.108288 (PMC9133570; doi:10.1016/j.dib.2022.108288)
Supplement: Supplementary file 1 [file mmc1.docx]

**Coping Strategies Questionnaire - German Version (CSQ-D) - not a validated translation**

Source: Verra ML, Angst F, Lehmann S et al. Translation, cross-cultural adaptation, reliability, and validity of the German version of the Coping Strategies Questionnaire (CSQ-D). J Pain 2006; 7 (5):327-36.

Name:_______________________________ birth date: ________________ date:________________

Individuals with pain have developed a variety of methods to cope or manage their pain. The following is a list of examples of pain management from patients interviewed. Please rate what you do when you are in pain. For each example, circle the number that applies to you. To do this, please use the scale of 0 - 6.

Note that you can circle any number in the scale that applies to you.

Example 1 When I am in pain, I take a break.

If you think that you often take a break when you are in pain, please circle 4:

| Never | Almost never | Rarely | Sometimes | Often | Most of the time | always |
| --- | --- | --- | --- | --- | --- | --- |
| 0 | 1 | 2 | 3 | 4 | 5 | 6 |

Example 2 When I have pain, I take a break.

If you think that you rarely take a break when you are in pain, please circle 2:

| Never | Almost never | Rarely | Sometimes | Often | Most of the time | always |
| --- | --- | --- | --- | --- | --- | --- |
| 0 | 1 | 2 | 3 | 4 | 5 | 6 |

Now please answer all the questions on the following pages, circling only the number that applies to you for each question.

When I have pain

1. I try to gain distance from the pain, almost as if the pain were in another person's body.

| Never | Almost never | Rarely | Sometimes | Often | Most of the time | always |
| --- | --- | --- | --- | --- | --- | --- |
| 0 | 1 | 2 | 3 | 4 | 5 | 6 |

1. I go out of the house and do something, such as going to the movies or shopping.

| Never | Almost never | Rarely | Sometimes | Often | Most of the time | always |
| --- | --- | --- | --- | --- | --- | --- |
| 0 | 1 | 2 | 3 | 4 | 5 | 6 |

1. I try to think of something pleasant pleasant things.

| Never | Almost never | Rarely | Sometimes | Often | Most of the time | always |
| --- | --- | --- | --- | --- | --- | --- |
| 0 | 1 | 2 | 3 | 4 | 5 | 6 |

1. I do not imagine the pain as pain, but rather as a dull or or warm feeling.

| Never | Almost never | Rarely | Sometimes | Often | Most of the time | always |
| --- | --- | --- | --- | --- | --- | --- |
| 0 | 1 | 2 | 3 | 4 | 5 | 6 |

1. It is terrible and I have the I feel like it never gets better.

| Never | Almost never | Rarely | Sometimes | Often | Most of the time | always |
| --- | --- | --- | --- | --- | --- | --- |
| 0 | 1 | 2 | 3 | 4 | 5 | 6 |

1. I tell myself to be brave, even though I am in pain.

| Never | Almost never | Rarely | Sometimes | Often | Most of the time | always |
| --- | --- | --- | --- | --- | --- | --- |
| 0 | 1 | 2 | 3 | 4 | 5 | 6 |

1. Reading.

| Never | Almost never | Rarely | Sometimes | Often | Most of the time | always |
| --- | --- | --- | --- | --- | --- | --- |
| 0 | 1 | 2 | 3 | 4 | 5 | 6 |

1. I tell myself that I can the pain.

| Never | Almost never | Rarely | Sometimes | Often | Most of the time | always |
| --- | --- | --- | --- | --- | --- | --- |
| 0 | 1 | 2 | 3 | 4 | 5 | 6 |

1. I take my medications.

| Never | Almost never | Rarely | Sometimes | Often | Most of the time | always |
| --- | --- | --- | --- | --- | --- | --- |
| 0 | 1 | 2 | 3 | 4 | 5 | 6 |

1. I count in my mind or think of a song.

| Never | Almost never | Rarely | Sometimes | Often | Most of the time | always |
| --- | --- | --- | --- | --- | --- | --- |
| 0 | 1 | 2 | 3 | 4 | 5 | 6 |

When I have pain:

1. I imagine the pain as another feeling, e.g. as numbness.

| Never | Almost never | Rarely | Sometimes | Often | Most of the time | always |
| --- | --- | --- | --- | --- | --- | --- |
| 0 | 1 | 2 | 3 | 4 | 5 | 6 |

1. It is terrible and I have the I have the feeling that the pain overwhelms me.

| Never | Almost never | Rarely | Sometimes | Often | Most of the time | always |
| --- | --- | --- | --- | --- | --- | --- |
| 0 | 1 | 2 | 3 | 4 | 5 | 6 |

1. I make mind games so that I not to think about the pain.

| Never | Almost never | Rarely | Sometimes | Often | Most of the time | always |
| --- | --- | --- | --- | --- | --- | --- |
| 0 | 1 | 2 | 3 | 4 | 5 | 6 |

1. I have the feeling that my life is no longer worth living.

| Never | Almost never | Rarely | Sometimes | Often | Most of the time | always |
| --- | --- | --- | --- | --- | --- | --- |
| 0 | 1 | 2 | 3 | 4 | 5 | 6 |

1. I know that one day someone will help me help me and the pain will go away go away for a while.

| Never | Almost never | Rarely | Sometimes | Often | Most of the time | always |
| --- | --- | --- | --- | --- | --- | --- |
| 0 | 1 | 2 | 3 | 4 | 5 | 6 |

1. I walk a lot.

| Never | Almost never | Rarely | Sometimes | Often | Most of the time | always |
| --- | --- | --- | --- | --- | --- | --- |
| 0 | 1 | 2 | 3 | 4 | 5 | 6 |

1. I pray to God that the pain does not last long.

| Never | Almost never | Rarely | Sometimes | Often | Most of the time | always |
| --- | --- | --- | --- | --- | --- | --- |
| 0 | 1 | 2 | 3 | 4 | 5 | 6 |

1. I try to imagine that the pain does not belong to my body but is rather outside of me.

| Never | Almost never | Rarely | Sometimes | Often | Most of the time | always |
| --- | --- | --- | --- | --- | --- | --- |
| 0 | 1 | 2 | 3 | 4 | 5 | 6 |

1. Relaxing.

| Never | Almost never | Rarely | Sometimes | Often | Most of the time | always |
| --- | --- | --- | --- | --- | --- | --- |
| 0 | 1 | 2 | 3 | 4 | 5 | 6 |

1. I do not think about the pain.

| Never | Almost never | Rarely | Sometimes | Often | Most of the time | always |
| --- | --- | --- | --- | --- | --- | --- |
| 0 | 1 | 2 | 3 | 4 | 5 | 6 |

1. I try to think about the coming years years to come and how everything will be I get rid of the pain.

| Never | Almost never | Rarely | Sometimes | Often | Most of the time | always |
| --- | --- | --- | --- | --- | --- | --- |
| 0 | 1 | 2 | 3 | 4 | 5 | 6 |

1. I tell myself that it doesn't hurt.

| Never | Almost never | Rarely | Sometimes | Often | Most of the time | always |
| --- | --- | --- | --- | --- | --- | --- |
| 0 | 1 | 2 | 3 | 4 | 5 | 6 |

1. I tell myself I can't let the pain pain get in the way of what I have get in the way of what I have to do.

| Never | Almost never | Rarely | Sometimes | Often | Most of the time | always |
| --- | --- | --- | --- | --- | --- | --- |
| 0 | 1 | 2 | 3 | 4 | 5 | 6 |

1. I do not pay attention to the pain.

| Never | Almost never | Rarely | Sometimes | Often | Most of the time | always |
| --- | --- | --- | --- | --- | --- | --- |
| 0 | 1 | 2 | 3 | 4 | 5 | 6 |

1. I have confidence in the doctors that my pain will be cured will be cured one day.

| Never | Almost never | Rarely | Sometimes | Often | Most of the time | always |
| --- | --- | --- | --- | --- | --- | --- |
| 0 | 1 | 2 | 3 | 4 | 5 | 6 |

1. No matter how bad the pain gets I know how to deal with it.

| Never | Almost never | Rarely | Sometimes | Often | Most of the time | always |
| --- | --- | --- | --- | --- | --- | --- |
| 0 | 1 | 2 | 3 | 4 | 5 | 6 |

1. I pretend the pain is not there.

| Never | Almost never | Rarely | Sometimes | Often | Most of the time | always |
| --- | --- | --- | --- | --- | --- | --- |
| 0 | 1 | 2 | 3 | 4 | 5 | 6 |

1. I worry if the pain will ever will ever stop.

| Never | Almost never | Rarely | Sometimes | Often | Most of the time | always |
| --- | --- | --- | --- | --- | --- | --- |
| 0 | 1 | 2 | 3 | 4 | 5 | 6 |

1. Lying down.

| Never | Almost never | Rarely | Sometimes | Often | Most of the time | always |
| --- | --- | --- | --- | --- | --- | --- |
| 0 | 1 | 2 | 3 | 4 | 5 | 6 |

1. I think back to beautiful experiences.

| Never | Almost never | Rarely | Sometimes | Often | Most of the time | always |
| --- | --- | --- | --- | --- | --- | --- |
| 0 | 1 | 2 | 3 | 4 | 5 | 6 |

When I have pain:

1. I think of people with whom I like to do something with.

| Never | Almost never | Rarely | Sometimes | Often | Most of the time | always |
| --- | --- | --- | --- | --- | --- | --- |
| 0 | 1 | 2 | 3 | 4 | 5 | 6 |

1. I pray for the pain to stop.

| Never | Almost never | Rarely | Sometimes | Often | Most of the time | always |
| --- | --- | --- | --- | --- | --- | --- |
| 0 | 1 | 2 | 3 | 4 | 5 | 6 |

1. I take a shower or a bath.

| Never | Almost never | Rarely | Sometimes | Often | Most of the time | always |
| --- | --- | --- | --- | --- | --- | --- |
| 0 | 1 | 2 | 3 | 4 | 5 | 6 |

1. I imagine that the pain is is outside my body.

| Never | Almost never | Rarely | Sometimes | Often | Most of the time | always |
| --- | --- | --- | --- | --- | --- | --- |
| 0 | 1 | 2 | 3 | 4 | 5 | 6 |

1. I continue as if nothing happened.

| Never | Almost never | Rarely | Sometimes | Often | Most of the time | always |
| --- | --- | --- | --- | --- | --- | --- |
| 0 | 1 | 2 | 3 | 4 | 5 | 6 |

1. I see pain as a challenge challenge and do not allow it to disturb me.

| Never | Almost never | Rarely | Sometimes | Often | Most of the time | always |
| --- | --- | --- | --- | --- | --- | --- |
| 0 | 1 | 2 | 3 | 4 | 5 | 6 |

1. Even though it hurts, I just on.

| Never | Almost never | Rarely | Sometimes | Often | Most of the time | always |
| --- | --- | --- | --- | --- | --- | --- |
| 0 | 1 | 2 | 3 | 4 | 5 | 6 |

1. I have the feeling that I can't take the pain anymore.

| Never | Almost never | Rarely | Sometimes | Often | Most of the time | always |
| --- | --- | --- | --- | --- | --- | --- |
| 0 | 1 | 2 | 3 | 4 | 5 | 6 |

1. Trying to socialize.

| Never | Almost never | Rarely | Sometimes | Often | Most of the time | always |
| --- | --- | --- | --- | --- | --- | --- |
| 0 | 1 | 2 | 3 | 4 | 5 | 6 |

1. I ignore the pain.

| Never | Almost never | Rarely | Sometimes | Often | Most of the time | always |
| --- | --- | --- | --- | --- | --- | --- |
| 0 | 1 | 2 | 3 | 4 | 5 | 6 |

1. I rely on my faith in God.

| Never | Almost never | Rarely | Sometimes | Often | Most of the time | always |
| --- | --- | --- | --- | --- | --- | --- |
| 0 | 1 | 2 | 3 | 4 | 5 | 6 |

1. I have the feeling that I cannot Able to do more.

| Never | Almost never | Rarely | Sometimes | Often | Most of the time | always |
| --- | --- | --- | --- | --- | --- | --- |
| 0 | 1 | 2 | 3 | 4 | 5 | 6 |

1. I think about things that I like to do.

| Never | Almost never | Rarely | Sometimes | Often | Most of the time | always |
| --- | --- | --- | --- | --- | --- | --- |
| 0 | 1 | 2 | 3 | 4 | 5 | 6 |

1. I do everything to distract myself from the pain from the pain.

| Never | Almost never | Rarely | Sometimes | Often | Most of the time | always |
| --- | --- | --- | --- | --- | --- | --- |
| 0 | 1 | 2 | 3 | 4 | 5 | 6 |

1. I am doing something enjoyable, such as watching TV or listening to music.

| Never | Almost never | Rarely | Sometimes | Often | Most of the time | always |
| --- | --- | --- | --- | --- | --- | --- |
| 0 | 1 | 2 | 3 | 4 | 5 | 6 |

1. I imagine that the pain is not a part of me. part of me.

| Never | Almost never | Rarely | Sometimes | Often | Most of the time | always |
| --- | --- | --- | --- | --- | --- | --- |
| 0 | 1 | 2 | 3 | 4 | 5 | 6 |

1. I do something active, such as. household chores or other everyday activities.

| Never | Almost never | Rarely | Sometimes | Often | Most of the time | always |
| --- | --- | --- | --- | --- | --- | --- |
| 0 | 1 | 2 | 3 | 4 | 5 | 6 |

1. I use a heating pad.

| Never | Almost never | Rarely | Sometimes | Often | Most of the time | always |
| --- | --- | --- | --- | --- | --- | --- |
| 0 | 1 | 2 | 3 | 4 | 5 | 6 |

Because of all your methods of dealing with, or coping with, pain:

1. How much control do you have an average day over your pain?

| Not at all | Very little | Little | Medium-strong | Strong | Very strong | Full-permanent |
| --- | --- | --- | --- | --- | --- | --- |
| 0 | 1 | 2 | 3 | 4 | 5 | 6 |

1. How much can you reduce pain on an average day? reduce?

| Not at all | Very little | Little | Medium-strong | Strong | Very strong | Full-permanent |
| --- | --- | --- | --- | --- | --- | --- |
| 0 | 1 | 2 | 3 | 4 | 5 | 6 |

Please check again if you have answered all questions.
