# Supplementary material for: Data on pain coping strategies and their association with quality of life in people with Parkinson’s disease: A cross-sectional study
Source: Data Brief. 2022 May 17;42:108288. doi: 10.1016/j.dib.2022.108288 (PMC9133570; doi:10.1016/j.dib.2022.108288)
Supplement: Supplementary file 2 [file mmc2.pdf]

Coping-Strategies-Questionnaire – Deutsche Version (CSQ-D)

Quelle: Verra ML, Angst F, Lehmann S et al. Translation, cross-cultural adaptation, reliability, and validity of the German version of the Coping Strategies Questionnaire (CSQ-D). J Pain 2006; 7 (5):327-36.

Name:________________________________ Geburtsdatum: ________________ Datum:________________

Personen mit Schmerzen haben eine Vielzahl von Methoden entwickelt, mit ihren Schmerzen fertig zu werden oder mit ihnen umzugehen. Es folgt eine Liste mit Beispielen der Schmerzbewältigung von befragten Patienten. Bitte beurteilen Sie, was Sie machen, wenn Sie Schmerzen haben. Bei jedem Beispiel umkreisen Sie die für Sie zutreffende Zahl. Dazu benutzen Sie bitte die Skala von 0 - 6.
Beachten Sie, dass Sie irgendeine für Sie zutreffende Zahl in der Skala umkreisen können.

Beispiel 1 Wenn ich Schmerzen habe, mache ich eine Pause.

	**nie**	**fast nie**	**selten**	**manch-mal**	**oft**	**meis-tens**	**immer**
Wenn Sie der Meinung sind, dass Sie oft eine Pause machen, wenn Sie Schmerzen haben, umkreisen Sie bitte die 4:	**0**	**1**	**2**	**3**	**(4)**	**5**	**6**

Beispiel 2 Wenn ich Schmerzen habe, mache ich eine Pause.

	**nie**	**fast nie**	**selten**	**manch-mal**	**oft**	**meis-tens**	**immer**
Wenn Sie der Meinung sind, dass Sie selten eine Pause machen, wenn Sie Schmerzen haben, umkreisen Sie bitte die 2:	**0**	**1**	**(2)**	**3**	**4**	**5**	**6**

Bitte beantworten Sie nun *alle* Fragen auf den folgenden Seiten und umkreisen Sie für eine Frage jeweils *nur die Zahl, die auf Sie zutrifft*.

	**Wenn ich Schmerzen habe:**	**nie**	**fast nie**	**selten**	**manch-mal**	**oft**	**meistens**	**immer**
1.	Ich versuche, Abstand von den Schmerzen zu gewinnen, fast so, als ob die Schmerzen im Körper einer anderen Person wären.	0	1	2	3	4	5	6
2.	Ich gehe aus dem Haus und unternehme etwas, wie zum Beispiel ins Kino oder einkaufen gehen.	0	1	2	3	4	5	6
3.	Ich versuche, an etwas Angenehmes zu denken.	0	1	2	3	4	5	6
4.	Ich stelle den Schmerz mir nicht als Schmerz vor, sondern eher als dumpfes oder warmes Gefühl.	0	1	2	3	4	5	6
5.	Es ist schrecklich und ich habe das Gefühl, es wird nie besser.	0	1	2	3	4	5	6
6.	Ich sage mir, dass ich tapfer sein muss, obwohl ich Schmerzen habe.	0	1	2	3	4	5	6
7.	Ich lese.	0	1	2	3	4	5	6
8.	Ich sage mir, dass ich den Schmerz bezwingen kann.	0	1	2	3	4	5	6
9.	Ich nehme meine Medikamente ein.	0	1	2	3	4	5	6
10.	Ich zähle in Gedanken oder denke an ein Lied.	0	1	2	3	4	5	6

Aus: Oesch, Hilfiker, Keller, Kool, Luomajoki, Tal-Akabi, Schädler, Verra, Widmer Leu
Assessments in der Rehabilitation - Band 2: Bewegungsapparat. © Hogrefe Verlag 2017. Alle Rechte vorbehalten.

	**Wenn ich Schmerzen habe:**	**nie**	**fast nie**	**selten**	**manch-mal**	**oft**	**meis-tens**	**immer**
11.	Ich stelle mir den Schmerz als anderes Gefühl vor, z.B. als Taubheit.	0	1	2	3	4	5	6
12.	Es ist schrecklich und ich habe das Gefühl, dass der Schmerz mich überwältigt.	0	1	2	3	4	5	6
13.	Ich mache Gedankenspiele, damit ich nicht an den Schmerz denke.	0	1	2	3	4	5	6
14.	Ich habe das Gefühl, dass mein Leben nicht mehr lebenswert ist.	0	1	2	3	4	5	6
15.	Ich weiss, eines Tages wird mir jemand helfen und der Schmerz wird für eine Weile weggehen.	0	1	2	3	4	5	6
16.	Ich spaziere viel.	0	1	2	3	4	5	6
17.	Ich bete zu Gott, dass der Schmerz nicht lange anhält.	0	1	2	3	4	5	6
18	Ich versuche mir vorzustellen, dass der Schmerz nicht zu meinem Körper gehört, sondern eher ausserhalb von mir ist.	0	1	2	3	4	5	6
19.	Ich entspanne mich.	0	1	2	3	4	5	6
20.	Ich denke nicht an den Schmerz.	0	1	2	3	4	5	6
21.	Ich versuche, an die kommenden Jahre zu denken und wie alles sein wird, wenn ich den Schmerz losgeworden bin.	0	1	2	3	4	5	6
22.	Ich sage mir, dass es nicht weh tut.	0	1	2	3	4	5	6
23.	Ich sage mir, ich darf nicht zulassen, dass mir der Schmerz bei dem, was ich zu tun habe, im Wege steht.	0	1	2	3	4	5	6
24.	Ich beachte den Schmerz nicht.	0	1	2	3	4	5	6
25.	Ich habe Vertrauen in die Ärzte, dass meine Schmerzen eines Tages geheilt werden.	0	1	2	3	4	5	6
26.	Egal, wie schlimm die Schmerzen werden, ich weiss, wie ich damit umgehe.	0	1	2	3	4	5	6
27.	Ich tue so, als ob der Schmerz nicht da wä	0	1	2	3	4	5	6
28.	Ich mache mir Sorgen, ob der Schmerz jemals aufhören wird.	0	1	2	3	4	5	6
29.	Ich lege mich hin.	0	1	2	3	4	5	6
30.	Ich denke an schöne Erlebnisse zurück.	0	1	2	3	4	5	6

Aus: Oesch, Hilfiker, Keller, Kool, Luomajoki, Tal-Akabi, Schädler, Verra, Widmer Leu
Assessments in der Rehabilitation - Band 2: Bewegungsapparat. © Hogrefe Verlag 2017. Alle Rechte vorbehalten.

	**Wenn ich Schmerzen habe:**	**nie**	**fast nie**	**selten**	**manch -mal**	**oft**	**meis-tens**	**immer**
31.	Ich denke an Leute, mit denen ich gerne etwas unternehme.	0	1	2	3	4	5	6
32.	Ich bete, damit der Schmerz aufhört.	0	1	2	3	4	5	6
33.	Ich dusche oder nehme ein Bad.	0	1	2	3	4	5	6
34.	Ich stelle mir vor, dass der Schmerz ausserhalb meines Körpers ist.	0	1	2	3	4	5	6
35.	Ich mache weiter, als ob nichts geschehen wäre.	0	1	2	3	4	5	6
36.	Ich sehe den Schmerz als Heraus-Forderung und lasse nicht zu, dass er mich stört.	0	1	2	3	4	5	6
37.	Obwohl es weh tut, mache ich einfach weiter.	0	1	2	3	4	5	6
38.	Ich habe das Gefühl, dass ich den Schmerz nicht mehr aushalten kann.	0	1	2	3	4	5	6
39.	Ich versuche, unter Leute zu gehen.	0	1	2	3	4	5	6
40.	Ich ignoriere den Schmerz.	0	1	2	3	4	5	6
41.	Ich verlasse mich auf meinen Glauben an Gott.	0	1	2	3	4	5	6
42.	Ich habe das Gefühl, dass ich nicht Mehr kann.	0	1	2	3	4	5	6
43.	Ich denke an Dinge, die ich gerne mache.	0	1	2	3	4	5	6
44.	Ich mache alles, um mich vom Schmerz abzulenken.	0	1	2	3	4	5	6
45.	Ich mache etwas Erfreuliches, wie z.B. fernsehen oder Musik hören.	0	1	2	3	4	5	6
46.	Ich stelle mir vor, dass der Schmerz kein Teil von mir ist.	0	1	2	3	4	5	6
47.	Ich mache etwas Aktives, wie z.B. Haushaltarbeiten oder andere Alltagstätigkeiten.	0	1	2	3	4	5	6
48.	Ich benutze ein Heizkissen.	0	1	2	3	4	5	6

Aus: Oesch, Hilfiker, Keller, Kool, Luomajoki, Tal-Akabi, Schädler, Verra, Widmer Leu
Assessments in der Rehabilitation - Band 2: Bewegungsapparat. © Hogrefe Verlag 2017. Alle Rechte vorbehalten.

**Aufgrund all Ihrer Methoden, mit dem Schmerz umzugehen, oder damit fertig zu werden:**	**keine**	**fast keine**	**wenig**	**durch-schnitt-liche**	**starke**	**fast voll-ständige**	**voll-ständige**
49. **Wie viel Kontrolle** haben Sie an einem durchschnittlichen Tag über Ihre Schmerzen?	0	1	2	3	4	5	6
	**gar nicht**	**sehr wenig**	**wenig**	**mittel-stark**	**stark**	**sehr stark**	**voll-ständig**
50. **Wie stark** können Sie den Schmerz an einem durchschnittlichen Tag **reduzieren**?	0	1	2	3	4	5	6

Bitte kontrollieren Sie noch einmal, ob Sie alle Fragen beantwortet haben.

Aus: Oesch, Hilfiker, Keller, Kool, Luomajoki, Tal-Akabi, Schädler, Verra, Widmer Leu
Assessments in der Rehabilitation - Band 2: Bewegungsapparat. © Hogrefe Verlag 2017. Alle Rechte vorbehalten.
